# Network-Based Pharmacological Study on the Mechanism of Action of Buxue Liqi Huatan Decoction in the Treatment of Lung Cancer

**DOI:** 10.1155/2022/3418687

**Published:** 2022-08-19

**Authors:** Huabing Wei, Lihuang Zhou, Xiaojing Zhao, Feng Xie

**Affiliations:** Department of Thoracic Surgery, Renji Hospital, School of Medicine, Shanghai Jiao Tong University, Shanghai, China

## Abstract

**Objective:**

This study aimed to investigate the mechanism of action of Buxue Liqi Huatan decoction against lung cancer through network pharmacology.

**Methods:**

The chemical composition and targets of all the drugs in the Buxue Liqi Huatan decoction were obtained through the Database and Systematic Analysis Platform of Traditional Chinese Medicine Pharmacology, the Integrated Database of Traditional Chinese Medicine, and by screening lung cancer targets with the gene map and OMIM database. The targets were then imported into Cytoscape 3.7.2 to build a target network of active ingredients and imported into the STRING database to build a protein-protein interaction network. The BisoGenet plug-in in Cytoscape 3.7.2 was used for network topology analysis. Genetic ontology (GO) enrichment analysis and Kyoto Encyclopedia of Gene and Genomes (KEGG) enrichment analysis were performed on potential targets of the Buxue Liqi Huatan decoction for lung cancer using the R-language Bioconductor platform, and results were imported from Cytoscape 3.7.2 to obtain the KEGG network connection diagram via the Autodock molecular docking software.

**Results:**

A total of 238 chemical components and 694 disease targets were obtained, including 133 intersecting targets. The key targets included TP53, AKT1, and MYC, and the GO functional analysis was mainly related to oxidative and cellular oxidative stress, apoptotic signaling, and antibiotic response. The results showed that the key target with the best binding performance was TP53.

**Conclusion:**

The treatment of lung cancer with blood-supplementing, qi-transforming, and phlegm-transforming soups works through multiple components and targets. The active ingredients include quercetin, luteolin, naringenin, and baicalein. Among them, the core proteins of PPI protein interaction mainly include TP53, AKT1, MYC, EGRF, CCNB1, and ESR1. The enrichment analysis results show that the TNF signal pathway, PI3K-Akt signal pathway, AGE-RAGE, IL-17, etc., are the main signal pathways of Buxue Liqi Huatan decoction in treating lung cancer. This lays the foundation for further study of its mechanism.

## 1. Introduction

Lung cancer is one of the most common and fatal malignant tumors in China [1], and the five-year survival rate of patients with lung cancer is only 19% [2]. According to statistics, in 2018, more than 2.9 million new cases of lung cancer were reported worldwide, and the death toll from lung cancer reached more than 1.76 million. The incidence of lung cancer in men is higher than that in women, and it is higher in urban areas than in rural areas; therefore, the incidence and mortality rates have significant regional differences [3]. The surgical resection and chemotherapy are often used for the clinical treatment of lung cancer; however, the quality of life of the patient becomes low after these. Traditional Chinese Medicine (TCM) treatment based on syndrome differentiation plays an important role in the management of precancerous lesions and the prevention of tumor metastasis and multidrug resistance [4].

Network pharmacology explains the occurrence and development of diseases from the perspectives of system biology and biological network balance, recognizes the interactions between drugs, and guides the discovery of new drugs to improve or restore balance to the biological network of the body. In recent years, an increasing number of researchers have explored the relationship between composite multielement targets and diseases from the perspective of system biology, thereby providing a new direction for modern TCM research [5]. Therefore, in this study, the pharmacology of the network was used as a breakthrough to analyze the target action of Buxue Liqi Huatan decoction in the treatment of lung cancer and predict the possible mechanisms underlying its use.

## 2. Materials and Methods

### 2.1. Screening of Target Components of Buxue Liqi Huatan Decoction

The active chemical components of Chinese medicines (*Rhizoma Pinelliae*, *Rhizoma Arisaematis*, *Tianlong*, *Scorpio*, *Pericarpium Citri Reticulate*, *Endothelium Corneum Gigeriae Galli*, and *Radix Glycyrrhizae Preparata)* in the Buxue Liqi Huatan decoction were searched through TCM system pharmacology platforms: Traditional Chinese Medicine Pharmacology (TCMSP) and the Integrated Database of Traditional Chinese Medicine (TCMID). TCMSP was selected based on these settings: oral biologics (OB) ≥30% and drug dependence (DL) ≥0.18 [5]. TCMID was screened using SwissADME. The screening conditions were as follows: a high gastrointestinal (GI) absorption rate (pharmacokinetics) and more than two “yes” in drug-likeness. Ineffective components were removed, and the active components of Chinese medicine in the Buxue Liqi Huatan decoction were obtained. Potential proteins were obtained through the Swiss forecast of the objectives database with a screening condition of a probability ≥0.1, and the screened protein targets were converted to standardized gene names in the UniProt database.

### 2.2. Screening of Lung Cancer Disease-Related Targets

Lung cancer-related target genes were obtained using “gastric cancer” as the search term in the OMIM and GeneCards databases. There were too many targets retrieved from the GeneCards database, and the number of targets was filtered based on score values. A larger score value indicated that the target had a stronger correlation with the disease. Based on the assumption that a target with a score value greater than the median was the potential target for the disease, genes with scores greater than 5 that were left behind in the GeneCards database were merged and deduplicated in the OMIM database, that is, the target genes that were related to lung cancer disease.

### 2.3. Acquisition of Effective Targets and Venn Diagram

A Venn diagram was used to determine the intersection of the target of the Buxue Liqi Huatan decoction and the target of lung cancer to obtain the intersection target of the two, which was the effective target of Buxue Liqi Huatan decoction in the treatment of lung cancer.

### 2.4. Analysis of Active Ingredient-Effective Target Network Construction Analysis

Drug active components and effective target genes were imported into Cytoscape 3.7.2 software for networking and visual analysis [6], and a drug active components-target network diagram was obtained. The importance of the drug active components and their action targets was evaluated by analyzing network topology parameters, such as the degree value.

### 2.5. Protein Network Construction

The effective targets of Buxue Liqi Huatan decoction and lung cancer were imported into the STRING database to create a protein interaction network, and data with a reliability ≥0.900 were selected. Results were imported into Cytoscape 3.7.2 software for visual analysis in the tsv format. Protein-protein interaction (PPI) data were imported using R software to learn the connection points of core genes [6], and a histogram showing the top 30 core genes was obtained.

### 2.6. Enrichment Analysis of Target Function and Pathway

The software R (https://www.r-project.org/) and its base database were used to obtain the genetic ID (entrezid) of the potential action point. Other programs such as DOS, Cluster Profiler, and others were used as well. The Pathview (bioconductor) software package was used to analyze the GO function of these potential targets, including biological process (BP), cell component (CC), and molecular function (MF) analyses. The *p* value cutoff was 0, and the *q*-value cutoff was 0.05. GO concentration analysis was divided into three categories: BP, MF, and CC. Each category was classified according to the degree of importance, and the first 10 rich items are presented in a histogram and bubble chart.

### 2.7. Main Active Components-Target Molecular Docking of Buxue Liqi Huatan Decoction

The targets of Buxue Liqi Huatan decoction on lung cancer were searched in the protein data bank (PDB) database and saved in PDB format. The ligands were stored in the mol2 format as the top 2 compounds by degree after topological analysis. The potential targets of Buxue Liqi Huatan decoction on lung cancer were molecularly docked with the main compounds in Buxue Liqi Huatan decoction using AutoDockTools 1.5.6.

## 3. Results

### 3.1. Acquisition of Active Components and Related Objectives of the Buxue Liqi Huatan Decoction

In TCMSP and TCMID, 238 active components in Buxue Liqi Huatan decoction were obtained using OB ≥30% and DL ≥0.18, a high GI absorption (pharmacokinetics), and more than two “yes” in drug-likeness as the screening conditions. The active components are shown in the supporting file (Table S1, supporting information). A total of 654 Chinese medicine targets were retrieved through TCMSP and TCMID.

### 3.2. Acquisition of Lung Cancer-Related Targets

Disease genes were obtained from GeneCards and OMIM databases. Based on experience, targets with scores greater than the median were set as potential disease targets, and the related targets retrieved from the OMIM database were combined, followed by the deletion of duplicate values. Finally, 694 lung cancer-related targets were obtained.

### 3.3. Venn Drawing

**Figure 1:**
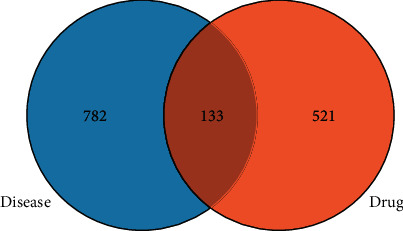
Venn diagram of the intersection target of Buxue Liqi Huatan decoction and lung cancer.

The Venn tool in TBtools was used to intersect the targets of Buxue Liqi Huatan decoction with those of lung cancer, and 133 intersecting targets of the two were obtained, with the result shown in Figure 1.

### 3.4. Construction of Network Diagram of Active Components-Effective Targets of Buxue Liqi Huatan Decoction

**Figure 2:**
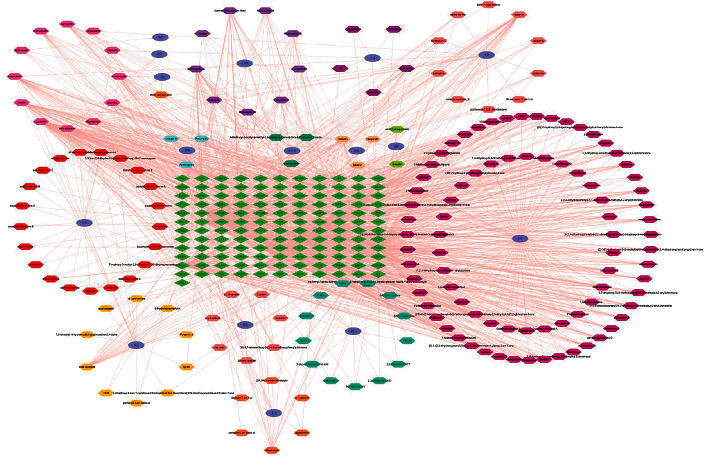
Network diagram of active components and effective targets of Buxue Liqi Huatan decoction.

The active components and effective target network of Buxue Liqi Huatan decoction were constructed using Cytoscape 3.7.2, as shown in Figure 2. The topological parameters of the network of Buxue Liqi Huatan decoction for the treatment of lung cancer were calculated using the software, to assess the importance of active components and action objectives. Results showed that the active components, such as *quercetin*, *luteolin,* and *naringenin,* could act on multiple targets, and these components might be the main active components of Buxue Liqi Huatan decoction in the treatment of lung cancer.

### 3.5. Protein Network Construction

**Figure 3:**
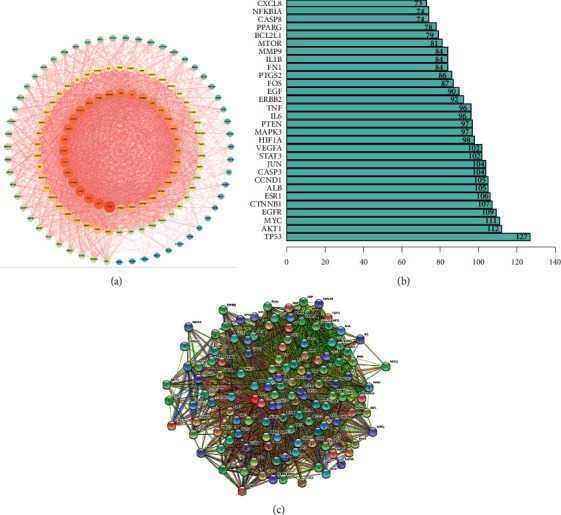
PPI network diagram of intersection targets. (a) PPI network diagram, (b) core gene map, and (c) protein mutual mapping.

The Venn tool in TBtools was used to intersect the target of Buxue Liqi Huatan decoction with the target of lung cancer, as shown in Figure 1. The intersected target was uploaded to the STRING database, and the setting credibility of ≥0.9 was used to obtain a target PPI network diagram. Data were imported into Cytoscape 3.7.2 to draw a protein network diagram. Larger degree values correspond to larger nodes. The location in the network was determined based on the degree value. According to Figure 3, the targets in the network center were TP53, AKT1, and MYC, which were presumed to be important targets of Buxue Liqi Huatan decoction for the treatment of lung cancer.

### 3.6. Results of Enrichment Analysis of Target Function and Pathway

**Figure 4:**
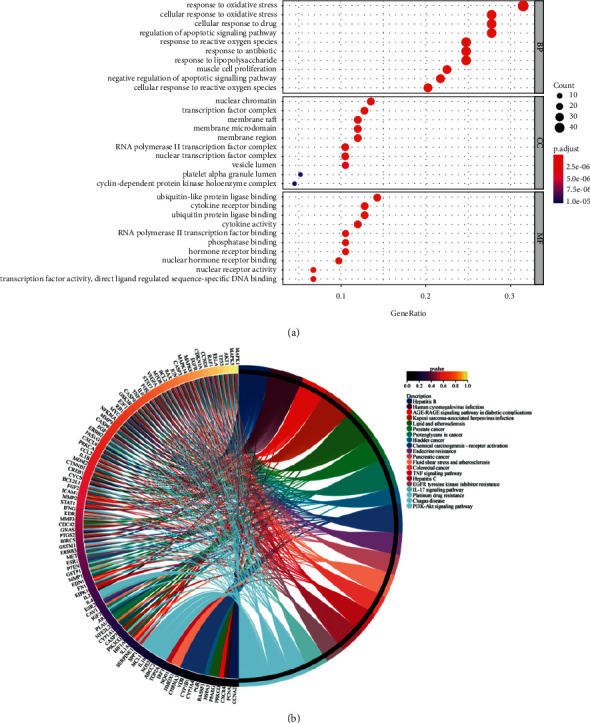
Analysis of enrichment of lung cancer treated with Buxue Liqi Huatan decoction. (a) GO enrichment analysis and (b) KEGG enrichment analysis circle.

Using R to perform GO annotation analysis on effective targets, we selected the top 10 results from BP, CC, and MF and found that BP was primarily involved in oxidative stress, cell oxidative stress, apoptosis signal, antibiotic response, and other aspects. MF is primarily involved in ubiquitination protein ligase binding, cytokine corresponding binding, phosphorylation site binding, etc. CC is primarily associated with nuclear chromatin, membrane rafts, transcription factor complexes, and the microfiltration zone. The results are shown in Figure 4(a). After KEGG enrichment analysis, 154 pathways were identified, including the AGE-RAGE, tumor necrosis factor (TNF), P13K-Akt, and IL-17 pathways. The first 20 pathways were selected and visualized, and the results are shown in Figure 4(b).

### 3.7. Molecular Docking Results of the Active Components in Buxue Liqi Huatan Decoction

**Figure 5:**
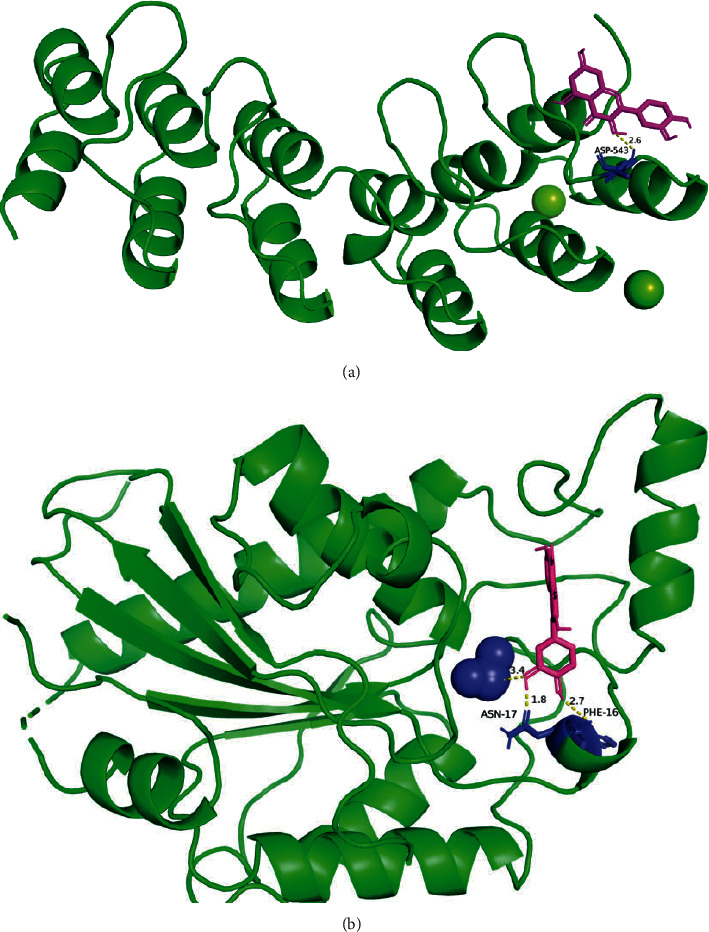
Molecular docking mode diagram. (a) Docking diagram of quercetin and AKT1 and (b) docking diagram of quercetin and TP53.

AutoDockTools 1.5.6 was used to conduct molecular coupling between potential targets of Buxue Liqi Huatan decoction on lung cancer and the main compounds after topology analysis and calculation. The more stable the structure of receptors and receptor combinations, the higher the possibility of occurrence. The top 2 targets were selected according to the degree value, namely, AKT1 and TP53 tumor protein. AutoDock Vina was used for molecular docking of the components, and a binding energy <−4.25 kcal/mol indicated that there was a certain binding activity between the small ligand molecule and the receptor protein. Binding energy <−5.0 kcal/mol indicated that there was good binding activity between them. Binding energy <−7.0 kcal/mol indicated that the ligand had a strong binding activity with the receptor [6]. The binding energies of quercetin core targets were −6.7 kcal/mol and −7.7 kcal/mol, indicating that the drug had a strong binding activity with the target paper. The specific docking results are shown in Figure 5.

## 4. Discussion

In this study, 238 active components were screened using network pharmacology, corresponding to 654 active component action targets, which were intersected with 915 gene targets. A total of 133 common targets were obtained, among which the active components included quercetin, luteolin, naringenin, and baicalein. The screened compounds were consistent with the results of existing studies, indicating that the screening results had a good reference value.

Quercetin has been proven to reduce oxidative stress, interfere with the renin-angiotensin system, and downregulate the reactive oxygen downstream signaling pathway to exert antioxidant, antitumor, anti-inflammatory, antibacterial, and cardiovascular protection as well as other pharmacological effects [7]. Studies have shown that luteolin has multitarget, multipathway, and multilink antitumor activities. Its mechanism of action includes inhibition of tumor cell proliferation, induction of tumor cell apoptosis, and inhibition of tumor metastasis [8]. A helmet can enhance the killing activity of natural killer cells on cancer cells, prevent the growth and migration of cancer cells, lead to the death of cancer cells, and hinder the glucose metabolism of cancer cells [9]. Baicalein can inhibit cell cycle progression and thus inhibit the proliferation of tumor cells by regulating the levels of different types of cyclins and cell cycle-dependent kinases [10]. In this study, we found that quercetin, luteolin, and baicalein could be connected to more protein targets and had good docking activity with the core targets, suggesting that they have potential anticancer activity. Among them, the core proteins for PPI were TP53, AKT1, MYC, eGFR, CCNB1, and ESR1. TP53 is a tumor suppressor gene, which is responsible for regulating the proliferation of tumor cells. The TP53 gene is an important anticancer gene, and its wild type can cause cancer cell apoptosis, thus preventing cancer, and it also has the function of helping cell genes repair defects [11, 12]. As an oncogene, AKT1 expression is upregulated in lung cancer. A study has revealed that the expression of AKT1 protein is related to the differentiation degree of lung cancer, lymph node metastasis, and TNM stage. A higher expression indicates that the lung cancer has a lower differentiation degree, a higher malignancy degree, and a worse prognosis, indicating that it is related to the procancer effect in the development of lung cancer [13]. Luteolin has a good binding ability to AKT1, can inhibit the proliferation, invasion, and migration of nonsmall cell lung cancer (NSCLC) A549 cells, and can induce apoptosis [14]. By regulating the expression of troponin and shrinking the cytoskeleton to reverse epithelial-mesenchymal transition (EMT), baicalein can inhibit the growth of lung cancer cells through the targeted Src/Id1 pathway. In addition, it can enhance the toxicity of cisplatin and enhance the sensitivity to chemotherapy, which may be related to EMT inhibition by blocking the intracellular Akt/NF-*κ*B signaling pathway [15]. The Myc gene was found in Burkitt lymphoma for the first time and can be activated by chromosome translocation [16]. Overexpression of myc is found in various tumors, such as lung cancer, stomach cancer, breast cancer, colon cancer, cervical cancer, some neuroblastomas, granulocytic leukemia, retinoblastoma, osteosarcoma, chondrosarcoma, chordoma, liposarcoma, rhabdomyosarcoma, Hodgkin's disease, and head tumors. All of them have amplification or overexpression of the myc gene. At least three Myc genes have been found, including C-myc, N-myc, and L-myc [17, 18].

The KEGG pathway enrichment analysis showed that TNF, PI3K-Akt, AGE-RAGE, and IL-17 signaling pathways were the main signaling pathways of Buxue Liqi Huatan decoction in the treatment of lung cancer. Among them, the PI3K-Akt signaling pathway plays an important role in tumor diseases [19, 20]. Overactivation of the PI3K/Akt signaling pathway leads to a decrease in the expression of tumor suppressor protein p53, which promotes the synthesis of protein, the proliferation of tumor cells, and inhibition of cell apoptosis [19]. Studies have found that the AGE/RAGE signaling pathway plays a role in the activation of EMT in cancer cells, and it can affect the dryness of cancer cells by regulating EMT. In addition, the PI3K/Akt signaling pathway can be used as the pathway between AGE/RAGE and EMT [21]. IL-17-mediated tumor angiogenesis is involved in the activation of the Stat3/GIV signaling pathway and subsequent upregulation of vascular endothelial growth factor production in NSCLC cells. This may be the mechanism underlying the correlation between IL-17, poor prognosis, and angiogenesis. Therapies targeting IL-17 and GIV can be developed as potential treatments to inhibit lung cancer [22, 23].

TNF is a central proinflammatory cytokine that is involved in a variety of inflammatory states, including autoimmunity. In 1975, Carswell et al. found that after BCG-vaccinated mice were injected with bacterial lipopolysaccharide, a substance appeared in the serum that could cause hemorrhagic necrosis of various tumors, and it was named tumor necrosis factor (TNF) [24]. TNF can kill some tumor cells in vivo and in vitro or inhibit proliferation. The sensitivity of tumor cell lines to TNF-*α* is very different, and TNF-*α* can even stimulate a few tumor cells [25, 26]. Treating tumor cells (such as mouse fibroblast L929) with actinomycin D, mitomycin C, and cycloheximide can obviously increase the killing activity of TNF-*α* on tumor cells. The response of tumors to TNF-*α* in vivo is also very different, which is not parallel to the sensitivity of tumor cell lines to TNF-*α* in vitro. There may be sensitive and resistant strains such as L929-S and L929-R in the same cell line. In addition, the expression of endogenous TNF in target cells may make cells resist the cytotoxic effect of exogenous TNF, so the sensitivity of cells to exogenous TNF can be changed by inducing or inhibiting the expression of endogenous TNF [27, 28]. Macrophage-binding TNF may be involved in killing target cells. TNF can affect various effector factors of immune monitoring and cause enhanced activity of natural killer cells and cytotoxic T lymphocytes [29]. TNF can also directly affect tumors and drive cells to respond to apoptosis, necrosis, survival, inflammation, or growth promotion. TNFR1 and TNFR2 are associated with various intracellular signaling pathways. TNFR1 can induce the expression of genes related to inflammation, survival, and proliferation by activating kinase signals and finally activating the nuclear factor NF-kB [30–32].

In summary, based on network pharmacology, we preliminarily explored the possible mechanisms underlying Buxue Liqi Huatan decoction in the treatment of lung cancer. The predicted action targets in this study are consistent with those of existing studies on Buxue Liqi Huatan decoction in the treatment of lung cancer, therefore reflecting the accuracy of target prediction. In addition, this study revealed that Buxue Liqi Huatan decoction treated lung cancer based on its multicomponent, multitarget, and multipathway activities and further provided the basis for experimental verification and clinical research. This study had some limitations. The targets obtained from different databases were not identical, so there might be discrepancies. Different screening criteria were adopted for data processing. Therefore, further experimental studies are required to verify and supplement the results of this study.

## Data Availability

The datasets used and analyzed in this study are available from the corresponding author upon reasonable request.

## Supplementary Materials

Supplementary Materials In the TCMSP and TCMID databases, 238 active components in Buxue Liqi Huatan decoction were obtained using OB ≥30% and DL ≥0.18, the active components are shown in Table S1. A total of 654 Chinese medicine targets were retrieved through TCMSP and TCMID databases.
