# Supplementary material for: Network-Based Pharmacological Study on the Mechanism of Action of Buxue Liqi Huatan Decoction in the Treatment of Lung Cancer
Source: Comput Intell Neurosci. 2022 Aug 19;2022:3418687. doi: 10.1155/2022/3418687 (PMC9417787; doi:10.1155/2022/3418687)
Supplement: Supplementary Materials — In the TCMSP and TCMID databases, 238 active components in Buxue Liqi Huatan decoction were obtained using OB ≥30% and DL ≥0.18, the active components are shown in Table S1. A total of 654 Chinese medicine targets were retrieved through TCMSP and TCMID databases. [file 3418687.f1.doc]

**Table S1 Active Components of Buxue Liqi Huatan Decoction**

| Mol ID | Molecule Name | OB(%) | DL |
| --- | --- | --- | --- |
| MOL000358 | beta-sitosterol | 36.91% | 0.75 |
| MOL000449 | Stigmasterol | 43.83% | 0.76 |
| MOL001002 | ellagic acid | 43.06% | 0.43 |
| MOL001918 | paeoniflorgenone | 87.59% | 0.37 |
| MOL001921 | Lactiflorin | 49.12% | 0.8 |
| MOL001924 | paeoniflorin | 53.87% | 0.79 |
| MOL001925 | paeoniflorin_qt | 68.18% | 0.4 |
| MOL002714 | baicalein | 33.52% | 0.21 |
| MOL002776 | Baicalin | 40.12% | 0.75 |
| MOL000359 | sitosterol | 36.91% | 0.75 |
| MOL004355 | Spinasterol | 42.98% | 0.76 |
| MOL000492 | (+)-catechin | 54.83% | 0.24 |
| MOL006990 | (1S,2S,4R)-trans-2-hydroxy-1,8-cineole-B-D-glucopyranoside | 30.25% | 0.27 |
| MOL006992 | (2R,3R)-4-methoxyl-distylin | 59.98% | 0.3 |
| MOL006994 | 1-o-beta-d-glucopyranosyl-8-o-benzoylpaeonisuffrone_qt | 36.01% | 0.3 |
| MOL006996 | 1-o-beta-d-glucopyranosylpaeonisuffrone_qt | 65.08% | 0.35 |
| MOL006999 | stigmast-7-en-3-ol | 37.42% | 0.75 |
| MOL007003 | benzoyl paeoniflorin | 31.14% | 0.54 |
| MOL007004 | Albiflorin | 30.25% | 0.77 |
| MOL007005 | Albiflorin_qt | 48.70% | 0.33 |
| MOL007008 | 4-ethyl-paeoniflorin_qt | 56.87% | 0.44 |
| MOL007012 | 4-o-methyl-paeoniflorin_qt | 56.70% | 0.43 |
| MOL007014 | 8-debenzoylpaeonidanin | 31.74% | 0.45 |
| MOL007016 | Paeoniflorigenone | 65.33% | 0.37 |
| MOL007018 | 9-ethyl-neo-paeoniaflorin A_qt | 64.42% | 0.3 |
| MOL007022 | evofolinB | 64.74% | 0.22 |
| MOL007025 | isobenzoylpaeoniflorin | 31.14% | 0.54 |
| MOL002883 | Ethyl oleate (NF) | 32.40% | 0.19 |
| MOL005043 | campest-5-en-3beta-ol | 37.58% | 0.71 |
| MOL001494 | Mandenol | 42.00% | 0.19 |
| MOL002135 | Myricanone | 40.60% | 0.51 |
| MOL002140 | Perlolyrine | 65.95% | 0.27 |
| MOL002151 | senkyunone | 47.66% | 0.24 |
| MOL002157 | wallichilide | 42.31% | 0.71 |
| MOL000433 | FA | 68.96% | 0.71 |
| MOL013381 | Marmin | 38.23% | 0.31 |
| MOL002341 | Hesperetin | 70.31% | 0.27 |
| MOL004328 | naringenin | 59.29% | 0.21 |
| MOL005828 | nobiletin | 61.67% | 0.52 |
| MOL001323 | Sitosterol alpha1 | 43.28% | 0.78 |
| MOL001328 | 2,3-didehydro GA70 | 63.29% | 0.5 |
| MOL001329 | 2,3-didehydro GA77 | 88.08% | 0.53 |
| MOL001339 | GA119 | 76.36% | 0.49 |
| MOL001340 | GA120 | 84.85% | 0.45 |
| MOL001342 | GA121-isolactone | 72.70% | 0.54 |
| MOL001343 | GA122 | 64.79% | 0.5 |
| MOL001344 | GA122-isolactone | 88.11% | 0.54 |
| MOL001348 | gibberellin 17 | 94.64% | 0.49 |
| MOL001349 | 4a-formyl-7alpha-hydroxy-1-methyl-8-methylidene-4aalpha,4bbeta-gibbane-1alpha,10beta-dicarboxylic acid | 88.60% | 0.46 |
| MOL001350 | GA30 | 61.72% | 0.54 |
| MOL001351 | Gibberellin A44 | 101.61% | 0.54 |
| MOL001352 | GA54 | 64.21% | 0.53 |
| MOL001353 | GA60 | 93.17% | 0.53 |
| MOL001355 | GA63 | 65.54% | 0.54 |
| MOL001358 | gibberellin 7 | 73.80% | 0.5 |
| MOL001360 | GA77 | 87.89% | 0.53 |
| MOL001361 | GA87 | 68.85% | 0.57 |
| MOL001368 | 3-O-p-coumaroylquinic acid | 37.63% | 0.29 |
| MOL001371 | Populoside_qt | 108.89% | 0.2 |
| MOL000296 | hederagenin | 36.91% | 0.75 |
| MOL000493 | campesterol | 37.58% | 0.71 |
| MOL001771 | poriferast-5-en-3beta-ol | 36.91% | 0.75 |
| MOL002680 | Flavoxanthin | 60.41% | 0.56 |
| MOL002694 | 4-[(E)-4-(3,5-dimethoxy-4-oxo-1-cyclohexa-2,5-dienylidene)but-2-enylidene]-2,6-dimethoxycyclohexa-2,5-dien-1-one | 48.47% | 0.36 |
| MOL002695 | lignan | 43.32% | 0.65 |
| MOL002698 | lupeol-palmitate | 33.98% | 0.32 |
| MOL002706 | Phytoene | 39.56% | 0.5 |
| MOL002707 | phytofluene | 43.18% | 0.5 |
| MOL002710 | Pyrethrin II | 48.36% | 0.35 |
| MOL002712 | 6-Hydroxykaempferol | 62.13% | 0.27 |
| MOL002717 | qt_carthamone | 51.03% | 0.2 |
| MOL002719 | 6-Hydroxynaringenin | 33.23% | 0.24 |
| MOL002721 | quercetagetin | 45.01% | 0.31 |
| MOL002757 | 7,8-dimethyl-1H-pyrimido[5,6-g]quinoxaline-2,4-dione | 45.75% | 0.19 |
| MOL002773 | beta-carotene | 37.18% | 0.58 |
| MOL000422 | kaempferol | 41.88% | 0.24 |
| MOL000006 | luteolin | 36.16% | 0.25 |
| MOL000953 | CLR | 37.87% | 0.68 |
| MOL000098 | quercetin | 46.43% | 0.28 |
| MOL001006 | poriferasta-7,22E-dien-3beta-ol | 42.98% | 0.76 |
| MOL012461 | 28-norolean-17-en-3-ol | 35.93% | 0.78 |
| MOL012485 | achyranthoside c_qt | 66.62% | 0.18 |
| MOL012505 | bidentatoside,ii_qt | 31.76% | 0.59 |
| MOL012537 | Spinoside A | 41.75% | 0.4 |
| MOL012542 | β-ecdysterone | 44.23% | 0.82 |
| MOL001454 | berberine | 36.86% | 0.78 |
| MOL001458 | coptisine | 30.67% | 0.86 |
| MOL000173 | wogonin | 30.68% | 0.23 |
| MOL002643 | delta 7-stigmastenol | 37.42% | 0.75 |
| MOL002897 | epiberberine | 43.09% | 0.78 |
| MOL003847 | Inophyllum E | 38.81% | 0.85 |
| MOL000785 | palmatine | 64.60% | 0.65 |
| MOL000085 | beta-daucosterol_qt | 36.91% | 0.75 |
| MOL001297 | trans-gondoic acid | 30.70% | 0.2 |
| MOL000392 | formononetin | 69.67% | 0.21 |
| MOL000906 | wenjine | 47.93% | 0.27 |
| MOL000915 | (1S,10S),(4S,5S)-germacrone-1(10),4-diepoxide | 30.48% | 0.18 |
| MOL000940 | bisdemethoxycurcumin | 77.38% | 0.26 |
| MOL001004 | pelargonidin | 37.99% | 0.21 |
| MOL004440 | Peimisine | 57.40% | 0.81 |
| MOL004443 | Zhebeiresinol | 58.72% | 0.19 |
| MOL004444 | Ziebeimine | 64.25% | 0.7 |
| MOL004446 | 6-Methoxyl-2-acetyl-3-methyl-1,4-naphthoquinone-8-O-beta-D-glucopyranoside | 33.31% | 0.57 |
| MOL004450 | Chaksine | 65.63% | 0.66 |
| MOL001558 | sesamin | 56.55% | 0.83 |
| MOL005360 | malkangunin | 57.71% | 0.63 |
| MOL005384 | suchilactone | 57.52% | 0.56 |
| MOL009361 | 13,15-Dideoxyaconitine | 34.67% | 0.25 |
| MOL009363 | tuberostemonine C | 55.34% | 0.74 |
| MOL009374 | 7-methoxy-3-methyl-2,5-dihydroxy-9,10-dihydrophenanthrene | 59.00% | 0.21 |
| MOL009377 | bisdehydroneotuberostemonine | 51.14% | 0.74 |
| MOL009379 | 2-oxostenine | 72.94% | 0.34 |
| MOL009380 | bisdehydrostemoninine | 38.51% | 0.73 |
| MOL009381 | bisdehydrostemoninine A | 62.64% | 0.68 |
| MOL009382 | bisdehydrostemoninine B | 46.05% | 0.64 |
| MOL009386 | 3,3'-bis-(3,4-dihydro-4-hydroxy-6-methoxy)-2H-1-benzopyran | 52.11% | 0.54 |
| MOL009387 | didehydrotuberostemonine | 51.91% | 0.74 |
| MOL009388 | dihydrostemoninine | 68.01% | 0.72 |
| MOL009394 | stemonamine | 45.19% | 0.35 |
| MOL009409 | oxystemoninine | 42.79% | 0.77 |
| MOL009411 | protostemotinine | 45.99% | 0.75 |
| MOL009414 | sessilifoliamide C | 65.87% | 0.2 |
| MOL009419 | sessilifoliamide H | 43.68% | 0.68 |
| MOL009422 | sessilifoline B | 58.81% | 0.29 |
| MOL009423 | sessilistemonamine A | 40.28% | 0.73 |
| MOL009424 | sessilistemonamine B | 40.64% | 0.73 |
| MOL009430 | stemonamide | 67.46% | 0.38 |
| MOL009431 | stemonine | 81.75% | 0.72 |
| MOL009433 | stemoninine B | 74.77% | 0.73 |
| MOL009434 | stemoninoamide | 66.70% | 0.33 |
| MOL009436 | stemotinine | 38.69% | 0.46 |
| MOL009441 | (3S,3'R,4'R,9S,9aS)-4'-hydroxy-3'-methyl-3-[(2S,4S)-4-methyl-5-oxooxolan-2-yl]spiro[1,2,3,5,6,7,8,9a-octahydropyrrolo[1,2-a]azepine-9,5'-oxolane]-2'-one | 85.52% | 0.38 |
| MOL001645 | Linoleyl acetate | 42.10% | 0.2 |
| MOL000354 | isorhamnetin | 49.60% | 0.31 |
| MOL004598 | 3,5,6,7-tetramethoxy-2-(3,4,5-trimethoxyphenyl)chromone | 31.97% | 0.59 |
| MOL004609 | Areapillin | 48.96% | 0.41 |
| MOL013187 | Cubebin | 57.13% | 0.64 |
| MOL004624 | Longikaurin A | 47.72% | 0.53 |
| MOL004628 | Octalupine | 47.82% | 0.28 |
| MOL004644 | Sainfuran | 79.91% | 0.23 |
| MOL004648 | Troxerutin | 31.60% | 0.28 |
| MOL004653 | (+)-Anomalin | 46.06% | 0.66 |
| MOL004702 | saikosaponin c_qt | 30.50% | 0.63 |
| MOL004718 | α-spinasterol | 42.98% | 0.76 |
| MOL000490 | petunidin | 30.05% | 0.31 |
| MOL001484 | Inermine | 75.18% | 0.54 |
| MOL001792 | DFV | 32.76% | 0.18 |
| MOL000211 | Mairin | 55.38% | 0.78 |
| MOL002311 | Glycyrol | 90.78% | 0.67 |
| MOL000239 | Jaranol | 50.83% | 0.29 |
| MOL002565 | Medicarpin | 49.22% | 0.34 |
| MOL002844 | Pinocembrin | 64.72% | 0.18 |
| MOL003656 | Lupiwighteone | 51.64% | 0.37 |
| MOL003896 | 7-Methoxy-2-methyl isoflavone | 42.56% | 0.2 |
| MOL000417 | Calycosin | 47.75% | 0.24 |
| MOL004805 | (2S)-2-[4-hydroxy-3-(3-methylbut-2-enyl)phenyl]-8,8-dimethyl-2,3-dihydropyrano[2,3-f]chromen-4-one | 31.79% | 0.72 |
| MOL004806 | euchrenone | 30.29% | 0.57 |
| MOL004808 | glyasperin B | 65.22% | 0.44 |
| MOL004810 | glyasperin F | 75.84% | 0.54 |
| MOL004811 | Glyasperin C | 45.56% | 0.4 |
| MOL004814 | Isotrifoliol | 31.94% | 0.42 |
| MOL004815 | (E)-1-(2,4-dihydroxyphenyl)-3-(2,2-dimethylchromen-6-yl)prop-2-en-1-one | 39.62% | 0.35 |
| MOL004820 | kanzonols W | 50.48% | 0.52 |
| MOL004824 | (2S)-6-(2,4-dihydroxyphenyl)-2-(2-hydroxypropan-2-yl)-4-methoxy-2,3-dihydrofuro[3,2-g]chromen-7-one | 60.25% | 0.63 |
| MOL004827 | Semilicoisoflavone B | 48.78% | 0.55 |
| MOL004828 | Glepidotin A | 44.72% | 0.35 |
| MOL004829 | Glepidotin B | 64.46% | 0.34 |
| MOL004833 | Phaseolinisoflavan | 32.01% | 0.45 |
| MOL004835 | Glypallichalcone | 61.60% | 0.19 |
| MOL004838 | 8-(6-hydroxy-2-benzofuranyl)-2,2-dimethyl-5-chromenol | 58.44% | 0.38 |
| MOL004841 | Licochalcone B | 76.76% | 0.19 |
| MOL004848 | licochalcone G | 49.25% | 0.32 |
| MOL004849 | 3-(2,4-dihydroxyphenyl)-8-(1,1-dimethylprop-2-enyl)-7-hydroxy-5-methoxy-coumarin | 59.62% | 0.43 |
| MOL004855 | Licoricone | 63.58% | 0.47 |
| MOL004856 | Gancaonin A | 51.08% | 0.4 |
| MOL004857 | Gancaonin B | 48.79% | 0.45 |
| MOL004860 | licorice glycoside E | 32.89% | 0.27 |
| MOL004863 | 3-(3,4-dihydroxyphenyl)-5,7-dihydroxy-8-(3-methylbut-2-enyl)chromone | 66.37% | 0.41 |
| MOL004864 | 5,7-dihydroxy-3-(4-methoxyphenyl)-8-(3-methylbut-2-enyl)chromone | 30.49% | 0.41 |
| MOL004866 | 2-(3,4-dihydroxyphenyl)-5,7-dihydroxy-6-(3-methylbut-2-enyl)chromone | 44.15% | 0.41 |
| MOL004879 | Glycyrin | 52.61% | 0.47 |
| MOL004882 | Licocoumarone | 33.21% | 0.36 |
| MOL004883 | Licoisoflavone | 41.61% | 0.42 |
| MOL004884 | Licoisoflavone B | 38.93% | 0.55 |
| MOL004885 | licoisoflavanone | 52.47% | 0.54 |
| MOL004891 | shinpterocarpin | 80.30% | 0.73 |
| MOL004898 | (E)-3-[3,4-dihydroxy-5-(3-methylbut-2-enyl)phenyl]-1-(2,4-dihydroxyphenyl)prop-2-en-1-one | 46.27% | 0.31 |
| MOL004903 | liquiritin | 65.69% | 0.74 |
| MOL004904 | licopyranocoumarin | 80.36% | 0.65 |
| MOL004905 | 3,22-Dihydroxy-11-oxo-delta(12)-oleanene-27-alpha-methoxycarbonyl-29-oic acid | 34.32% | 0.55 |
| MOL004907 | Glyzaglabrin | 61.07% | 0.35 |
| MOL004908 | Glabridin | 53.25% | 0.47 |
| MOL004910 | Glabranin | 52.90% | 0.31 |
| MOL004911 | Glabrene | 46.27% | 0.44 |
| MOL004912 | Glabrone | 52.51% | 0.5 |
| MOL004913 | 1,3-dihydroxy-9-methoxy-6-benzofurano[3,2-c]chromenone | 48.14% | 0.43 |
| MOL004914 | 1,3-dihydroxy-8,9-dimethoxy-6-benzofurano[3,2-c]chromenone | 62.90% | 0.53 |
| MOL004915 | Eurycarpin A | 43.28% | 0.37 |
| MOL004917 | glycyroside | 37.25% | 0.79 |
| MOL004924 | (-)-Medicocarpin | 40.99% | 0.95 |
| MOL004935 | Sigmoidin-B | 34.88% | 0.41 |
| MOL004941 | (2R)-7-hydroxy-2-(4-hydroxyphenyl)chroman-4-one | 71.12% | 0.18 |
| MOL004945 | (2S)-7-hydroxy-2-(4-hydroxyphenyl)-8-(3-methylbut-2-enyl)chroman-4-one | 36.57% | 0.32 |
| MOL004948 | Isoglycyrol | 44.70% | 0.84 |
| MOL004949 | Isolicoflavonol | 45.17% | 0.42 |
| MOL004957 | HMO | 38.37% | 0.21 |
| MOL004959 | 1-Methoxyphaseollidin | 69.98% | 0.64 |
| MOL004961 | Quercetin der. | 46.45% | 0.33 |
| MOL004966 | 3'-Hydroxy-4'-O-Methylglabridin | 43.71% | 0.57 |
| MOL000497 | licochalcone a | 40.79% | 0.29 |
| MOL004974 | 3'-Methoxyglabridin | 46.16% | 0.57 |
| MOL004978 | 2-[(3R)-8,8-dimethyl-3,4-dihydro-2H-pyrano[6,5-f]chromen-3-yl]-5-methoxyphenol | 36.21% | 0.52 |
| MOL004980 | Inflacoumarin A | 39.71% | 0.33 |
| MOL004985 | icos-5-enoic acid | 30.70% | 0.2 |
| MOL004988 | Kanzonol F | 32.47% | 0.89 |
| MOL004989 | 6-prenylated eriodictyol | 39.22% | 0.41 |
| MOL004990 | 7,2',4'-trihydroxy－5-methoxy-3－arylcoumarin | 83.71% | 0.27 |
| MOL004991 | 7-Acetoxy-2-methylisoflavone | 38.92% | 0.26 |
| MOL004993 | 8-prenylated eriodictyol | 53.79% | 0.4 |
| MOL004996 | gadelaidic acid | 30.70% | 0.2 |
| MOL000500 | Vestitol | 74.66% | 0.21 |
| MOL005000 | Gancaonin G | 60.44% | 0.39 |
| MOL005001 | Gancaonin H | 50.10% | 0.78 |
| MOL005003 | Licoagrocarpin | 58.81% | 0.58 |
| MOL005007 | Glyasperins M | 72.67% | 0.59 |
| MOL005008 | Glycyrrhiza flavonol A | 41.28% | 0.6 |
| MOL005012 | Licoagroisoflavone | 57.28% | 0.49 |
| MOL005013 | 18α-hydroxyglycyrrhetic acid | 41.16% | 0.71 |
| MOL005016 | Odoratin | 49.95% | 0.3 |
| MOL005017 | Phaseol | 78.77% | 0.58 |
| MOL005018 | Xambioona | 54.85% | 0.87 |
| MOL005020 | dehydroglyasperins C | 53.82% | 0.37 |
| MOL001689 | acacetin | 34.97% | 0.24 |
| MOL004580 | cis-Dihydroquercetin | 66.44% | 0.27 |
| MOL005996 | 2-O-methyl-3―O-β-D-glucopyranosyl platycogenate A | 45.15% | 0.25 |
| MOL006026 | dimethyl 2-O-methyl-3-O-a-D-glucopyranosyl platycogenate A | 39.21% | 0.25 |
| MOL006070 | robinin | 39.84% | 0.71 |
